# Subgrouping of Iranian children and adolescents based on cardiometabolic risk factors using latent class analysis: The CASPIAN-V study

**DOI:** 10.22088/cjim.11.4.370

**Published:** 2020

**Authors:** Abbas Abbasi-Ghahramanloo, Ramin Heshmat, Amir-Masood Rafiemanzelat, Kimia Ghaderi, Mohammad Esmaeil Motlagh, Zeinab Ahadi, Gita Shafiee, Armita Mahdavi-Gorabi, Mostafa Qorbani, Roya Kelishadi

**Affiliations:** 1 Department of Public Health, School of Health, Ardabil University of Medical Sciences, Ardabil, Iran; 2 Chronic Diseases Research Center, Endocrinology and Metabolism Population Sciences Institute, Tehran University of Medical Sciences, Tehran, Iran; 3 School of Medicine, Isfahan University of Medical Sciences, Isfahan, Iran; 4 Department of Pediatrics, Ahvaz Jundishapur University of Medical Sciences, Ahvaz, Iran; 5 Department of Basic and Clinical Research, Tehran Heart Center, Tehran University of Medical Sciences, Tehran, Iran; 6 Non-Communicable Diseases Research Center, Alborz University of Medical Sciences, Karaj, Iran; 7 Endocrinology and Metabolism Research Center, Endocrinology and Metabolism Clinical Sciences Institute, Tehran University of Medical Sciences, Tehran, Iran; 8 Child Growth and Development Research Center, Research Institute for Primordial Prevention of Non-communicable Disease, Isfahan University of Medical Sciences, Isfahan, Iran

**Keywords:** Cardiometabolic, Metabolic syndrome, Latent class analysis, Children and adolescents, Iran

## Abstract

**Background:**

Cardiometabolic syndrome indicates the clustering of several risk factors. The aims of this study were to identify the subgroups of the Iranian children and adolescents on the basis of the components of the cardio-metabolic syndrome and assess the role of demographic characteristics, socioeconomic status and lifestyle-related behaviors on the membership of participants in each latent class.

**Methods:**

This cross-sectional study was performed on 3730 Iranian students in 2015 using stratified cluster. All students in each class completed anonymous and structured questionnaires. Abdominal obesity, high triglyceride (TG), low high-density lipoprotein (HDL), high blood pressure (BP), high fasting blood sugar (FBS), high low-density lipoprotein (LDL), high cholesterol and obesity were used for assessing the pattern of cardio metabolic risk as a latent variable. Data analysis was performed using PROC LCA in SAS software.

**Results:**

Four latent classes were identified in this study; namely 1) healthy (59.6%), 2) low risk (20.4%), 3) moderate risk (13.7%) and 4) high risk (6.4%). Being a female (OR=0.59, 95% CI: 0.46-0.74), living in a rural area (OR=0.45, 95% CI;0.33-0.60), high screen time (OR=1.56, 95% CI:1.09-2.24), and parental obesity (OR=1.52, 95% CI: 1.18-1.95) were associated with moderate risk class. Only living in rural areas (OR=0.71, 95% CI; 0.51-0.99) was associated with high risk class.

**Conclusion:**

About 20% of the students are in the moderate risk and high risk classes. Design and implement interventions according to risk-based class that seem necessary by considering probably risk and protective factors for the prevention of complications of cardiometabolic syndrome.

Cardiometabolic syndrome indicates the clustering of the several risk factors that increased risk for various conditions and diseases such as cardiovascular disease, kidney dysfunction, abnormalities in stress-related protein synthesis and so on (1, 2). The growing prevalence of cardiometabolic syndrome components has raised concerns about metabolic consequences among children and adolescents (3). For example, obesity prevalence has increased in recent years among this group of individuals. There is evidence that obese children are in the higher risk of adult obesity, type 2 diabetes, hypertension and dyslipidemia (3-5). On the other hand, there is not any standard definition of metabolic syndrome for children and adolescence. As a result, using different definitions, the prevalence of metabolic syndrome in this stratum is not clear (6).

There are some prior variable-centered researches among Iranian children that reported the Mets prevalence. Based on different definitions, the prevalence of Mets among children has been reported between 1-22%. Reported range of this prevalence varies in different criteria as follows: National Cholesterol Education Program-Adult Treatment Panel III: 3-16%, International Diabetes Federation: 0-8%, American Heart Association; 4-9.5%, The National Health and Nutrition Examination Survey III; 1-18% and de Ferranti; 0-22% (7). 

Based on different definitions and composite nature, cardiometabolic syndrome remains a discussable and debatable entity. For example whether Mets is a single syndrome or a co-occurrence of different syndromes and situation carrying different risks remains an unanswered question (8). 

For this reason, there have been many debates on the underlying cause of the syndrome (9). Although some risk factors are evaluated in different variable-centered studies, there has been many discussions and arguments on the etiology of cardiometabolic syndrome. Previously some person-centered studies have been conducted to determine the underlying cause or causes in the world. Factor analysis, confirmatory factor analysis, and structural equation modeling are the designs implemented in these studies (10-13). These methods measure the association between all the observed and unobserved variables on a continuous scale, without identifying the veracity of their association or existence of any background group of conditions which could be the reason for the different manifestations (9). Latent class analysis (LCA) evaluates the associations between observed and unobserved categorical variables and classifies homogeneous individuals (14). 

LCA strives to identify separated profile of subjects based on their presentation of cardiometabolic components by considering this possibility that maybe there are meaningful different relationships among cardiometabolic components in different subgroups (15). LCA has been used relatively little in medical research other than in the area of mental health. This is according to the author’s knowledge the first attempt to use LCA to identify the cardiometabolic syndrome among Iranian children and adolescents. Based on the abovementioned background, the aims of this study were to (a) identify the subgroups of children and adolescents on the basis of components of cardiometabolic syndrome, (b) document the prevalence of each subgroup and (c) assess the role of physical activity, screen time, socioeconomic status, parental obesity as well as the demographic factors that may play in forming the classification. 

## Method

**Measures: **This cross-sectional survey of CASPIAN- V study was performed on 3730 students in 2015. The sample was selected through stratified cluster sampling, a sampling method. The study participants were selected from students aged 7-18 years old (from primary and secondary schools). In this study, the students of urban and rural areas of 30 provinces of Iran were recruited. According to the residence area and level of education, proportional to size sampling scheme was conducted in each province with consideration of equal sex ratio. More information regarding the methodology of this study was reported in another paper(16)

After identifying the eligible students, an expert team of trained health care professionals bega withn data gathering. After the informed consent was signed by the students and their parents, questionnaires were completed. The mentioned questionnaire derived from World Health Organization- Global School Students Health Survey (WHO-GSHS) has been translated into Farsi. The validity and reliability of the Persian version of questionnaires were confirmed in other studies (17). For assessing physical measurements, the research team used standard protocols. To measure, WC non-elastic tape has been used and this variable was measured between the lower border of the rib cage and iliac crest. 

Using the mercury sphygmomanometer with a defined and appropriate cut off size, blood pressure was measured on the right arm in a sitting position. For more precision, blood pressure was measured two times with 5-minintervals consideration. The mean of the two measurements was registered for final quantity of blood pressure. Abdominal obesity was defined as waist to height ratio ≥ 0.5. High fasting blood sugar (FBG) ≥ 100 mg/dl, high triglyceride (TG) ≥ 100 mg/dl, high total cholesterol (TC): >200 mg/dl, high LDL ≥ 110 mg/dl and low HDL < 40 mg/dl (except than 15-19 years old boys < 45 mg/dl) were considered as abnormal. Low physical activity is defined as having less than 30 min duration of exercises per day in the last week. Additionally, screen time was measured using a self-administered questionnaire. 

This questionnaire includes questions about the average number of hours/days spent on watching TV or VCDs, personal computer of electronic games (EG) all days of the week. The scores of 2 hours or more in a day was considered as “high” screen time. To assess the socioeconomic status of the participants, we used some questions including parental education, parents’ job, possessing a private car, school type (public/private), and having personal computer combined as unique index values were categorized as low; intermediate and high SES. Parental obesity was defined as BMI≥ 30 kg/m^2^. 

**Statistical analysis: **The LCA model was used to detect the subgroups of cardiometabolic syndrome components. According to this model, a number of observed or indicator variables were aggregated to represent a categorical latent variable. In this model, it is assumed that by considering measurement error, categories of latent variable could explain the correlation between indicator variables. By using several iterations, LCA compare the frequency of response patterns (observed response patterns and expected ones) for each identified latent class. As a result, LCA calculates some statistics that help to select the best model. These statistics are G2, Akaik information criterion (AIC), Bayesian information criterion (BIC). For these statistics, the smaller value shows better fit of the model. Finally a model with the smallest values of AIC or BIC might be selected. 

There is a problem in computing the p-value in LCA. When the degrees of freedom are large, the reference distribution for the G2 statistics is not known. In this situation, calculation of p-value is impossible. But when the degrees of freedom are smaller than 60, we can calculate the p-values. In this study, to perform LCA, eight dichotomous indicator variables were used for assessing the pattern of cardiometabolic risk factors as a latent variable. These observable variables were abdominal obesity, high TG, low HDL, high BP, high FBS, high LDL, high cholesterol and obesity. After selecting final model, age of the students, gender, region, low physical activity, high screen time, SES, and parental obesity were entered as covariates in the model. Simple statistical analysis was done using SPSS Version 16 and LCA was performed by PROC LCA in the SAS 9.2. 

## Results

The mean age of subjects was 12.44±3.07 (Max: 18, Min: 7) years. From all the participants, 1962 (52.6%) were males. Most of the participants (72.7%) were urban citizens. The prevalence of each indicator variable in assessing the pattern of cardio metabolic is shown in table 1. The data in table 1 shows that the prevalence of having low HDL is higher (29.6%) than other components and the prevalence of having high FBS is lower (4.2%) than others. As seen in this table, having low HDL and obesity had a significant association with gender of the participants. By considering eight dichotomous indicators, there were 256 possible responses (pattern=2^n^). We attempted to fit the LCA models with classes ranging from 1 to 10. For each LCA model, G2, AIC, and BIC are shown in table 2. Based on model selection criteria, the four latent class model was appropriate. Table 3 shows the results of this model. This table has three parts. The first part shows the prevalence of each latent class. The second and third part shows item response probabilities and odds ratio of covariates for membership in each latent class, respectively. The first section of table 3 indicates that latent class 1, healthy, includes almost 60% of subjects and latent class 4, high risk, includes 6.4% of them. The second section of table 3, contains the conditional probabilities of a “yes” response to components of cardiometabolic. To calculate a “No” response, it is needed to subtract the item-response probabilities from 1. 

In LCA, item response probabilities are used in the interpretation and labeling of each latent class. For labeling these classes, the larger item response probabilities have more weight. While item response probabilities greater than 0.5 are considered as large ones. In latent class 4, high risk, three components of cardiometabolic have high probability. Individuals in this latent class were likely to have high TG, high LDL, and high cholesterol. In contrast,students in latent class 1, healthy, were not engaged in any of the cardiometabolic components. Latent class 2 and 3 reflect different patterns of clustering of risk factors of cardiometabolic in comparison with latent classes 1 and 4. Students in latent class 2, low risk, had a high probability of having high TG. In this class, the probability of having low HDL is relatively high (almost 49%) but other variables have low probability. Latent class 3, moderate risk, shows different type of people. In this class, the probability of having **abdominal obesity** and obesity is high. 

For calculating the odds ratios, the first class, healthy, is considered as reference class. Table 4 shows the result of multinomial regression analysis. This table indicates that being female, decreases the odds of membership in classes 2 and 3 compared to class 1. Similarly, being a rural citizen decreases the odds of membership in classes 3 and 4, compared to class 1. This table shows that having low physical activity and higher level of SES (than low) have no significant effect on the membership of subjects in different classes compared to the first class. 

**Table 1:** Prevalence of cardiometabolic components among the Iranian adolescents

	**Total(n=3730)**		**Male(n=1962)**		**Female(n=1768)**		**P-value**
**Items**	**N**	**%**	**N**	**%**	**N**	**%**	
**Abdominal obesity**	756	20.3	411	20.9	345	19.5	0.277
**High TG**	1028	27.6	527	26.9	501	28.3	0.314
**Low HDL**	1105	29.6	647	33.0	458	25.9	<0.001
**High BP**	374	10.0	206	10.5	168	9.5	0.311
**High FBS**	157	4.2	93	4.7	64	3.6	0.089
**High LDL**	659	17.7	338	17.2	321	18.2	0.458
**High TC**	184	4.9	98	5.0	86	4.9	0.854
**Obesity**	408	10.9	246	12.5	162	9.2	0.001

BP: blood pressure; FBS; fasting blood sugar; TG; triglyceride; HLD; high density lipoprotein; LDL: low density lipoprotein; TC: total cholesterol. Obesity, BMI>95th; low HDL: <40 mg/dl (except in boys 15-19 y old, that cut-off was < 45 mg/dl); high LDL: >110 mg/dl; high TG: 150 mg/dl; high TC: > 200 mg/dl; elevated FBS > 100 mg/dl; high BP: >90th (adjusted by age, sex, height).

**Table 2:** Comparisons of LCA models with different latent classes based on model selection statistics

**Number of latent class **	**number of parameters estimates **	**G** ^2^	**df**	**AIC**	**BIC**	**Maximum log-likelihood **
1	8	1702.32	247	1718.32	1768.12	-11968.75
2	17	950.99	238	984.99	1090.80	-11593.09
3	26	357.86	229	409.86	571.69	-11296.52
4	35	199.35	220	269.35	487.20	-11217.27
5	44	152.03	211	240.03	513.89	-11193.61
6	41	137.04	202	243.04	572.93	-11186.11
7	62	108.79	193	232.79	618.69	-11171.99
8	71	97.29	184	239.29	681.21	-11166.24
9	80	89.95	175	249.95	747.88	-11162.57
10	89	77.11	166	255.11	809.06	-11156.15

*Note. LCA: Latent class analysis; AIC: Akaike information criterion; BIC: Bayesian information criterion. *

**Table 3:** The four latent class models of components of cardiometabolic risk factors

	**Latent class**			
	**Healthy**	**Low risk**	**Moderate risk**	**High risk**
**Latent class prevalence**	**0.596**	**0.204**	**0.137**	**0.064**
Item-response probabilities	Probability of a “Yes”*			
**Abdominal obesity**	0.084	0.084	0.906	0.177
**High TG**	0.013	0.969	0.266	0.530
**Low HDL**	0.247	0.488	0.310	0.110
**High BP**	0.088	0.065	0.208	0.096
**High FBS**	0.032	0.075	0.038	0.039
**High LDL**	0.127	0.092	0.147	0.970
**High cholesterol**	0.000	0.016	0.000	0.720
**Obesity**	0.020	0.026	0.635	0.081

*Note. The probability of a “No” response can be calculated by subtracting the item-response probabilities shown above from 1. *

* Item-response probabilities >0.5 in bold to facilitate interpretation.

BP: blood pressure; FBS; fasting blood sugar; TG; triglyceride; HLD; high density lipoprotein; LDL: low density lipoprotein; TC: total cholesterol. Obesity, BMI>95th; low HDL: <40 mg/dl (except in boys 15-19 y old, that cut-off was < 45 mg/dl); high LDL: >110 mg/dl; high TG: 150 mg/dl; high TC: > 200 mg/dl; elevated FBS > 100 mg/dl; high BP: >90th (adjusted by age, sex, height).

**Table 4:** Predictors of membership in latent classes of cardiometabolic risk factors

**Predictors**	**C1**	**C2**	**C3**	**C4**
	OR(95%CI)	OR(95%CI)	OR(95%CI)	OR(95%CI)
Age (year)	Reference	1.09(1.05-1.12)^*^	0.99(0.95-1.03)	0.97(0.93-1.02)
Gender(female)	Reference	0.68(0.57-0.81)^ *^	0.59(0.46-0.74)^ *^	0.86(0.64-1.15)
Region (rural)	Reference	0.88(0.73-1.06)	0.45(0.33-0.60)^ *^	0.71(0.51-0.99)^ *^
Low physical activity	Reference	0.95(0.79-1.13)	0.75(0.59-0.96)^ *^	1.14(0.85-1.55)
High screen time	Reference	1.28(0.96-1.70)	1.56(1.09-2.24)^ *^	0.63(0.33-1.22)
SES(high/ low)	Reference	0.85(0.72-1.03)	1.18(0.94-1.50)	1.11(0.82-1.50)
Parental obesity	Reference	0.99(0.81-1.21)	1.52(1.18-1.95)^ *^	0.72(0.49-1.04)

SES: socioeconomic status

*statistically significant

## Discussion

In this study, the authors attempted to find the subgroups of students on the basis of cardiometabolic syndrome components using LCA approach and they finally found four latent classes. The authors named these classes as follows: healthy, low risk, moderate risk and high risk. The probability of engaging in each component of cardiometabolic is quite low in the first class (i.e. healthy). In the next class (i. e. low risk), however, the probability of having high TG is high. In class 3 (i. e. moderate risk) the probability of having high **abdominal obesity and obesity is high. Finally in the last class (i. e. high risk) the probability of high TG, high cholesterol and high LDL is high. To the best of the author’s knowledge, there are only a few studies that have used LCA to detect the latent classes of cardiometabolic syndrome components. In addition, the researchers have used different criteria to identify the subgroups. For this reason, it is not possible to compare the finding of the present study with other similar studies exactly. At any rate, some of which will be discussed below: **

**Arguelles et al. found two distinct subgroups for metabolic syndrome in male and female subjects, which were labeled as non-Mets and Mets for both males and females (** 18 **). A previous study in Iran showed that there are four latent classes of metabolic syndrome components among Iranian adults. These classes are labeled as non-Mets (38.4%), low risk (18.6%), high risk (24.2%) and Mets (18.7%). The result of this study indicated that most of the participants were identified as high risk and Mets (** 15 **). Some clinicians believed that the determinants of cardio vascular disease have accumulation tendency. As a result, the risk of developing these diseases increases along with the increasing components clustering ability (** 19 **). The comparisons of the results of LCA among Iranian adults (** 15 **) and this study reveals this fact that there is a different pattern of metabolic syndrome among the adults and children in Iran. Our results indicated that although the prevalence of** having low HDL is higher (29.6%) than other components among study participants, but this variable has no role in the classification of the students in LCA. In other words, in all four latent classes, the probability of having low HDL is low. On the other hand, having a high TG has an important role in classifying the subjects. Having high TG has high probability in two latent classes. Among the Iranian adults, abdominal obesity had high probability in three latent classes and reported as the most important variable in classifying the participants (15). In this study, abdominal obesity and obesity co-occurs with each other and these indicators have high probability only in the moderate risk class with 13.7% prevalence. **From prevention view, based on the finding of the present study, it should pay attention in the reduction of TG level among the Iranian children. **

**Considering co-occurrence and clustering pattern with co-changing nature of cardiometabolic syndrome, could be an effective approach in the prevention programs. We examined this pattern by LCA and were able to classify the students into four sub classes. It is very important to say that we could not show the pathophysiological mechanism of cardiometabolic syndrome but the co-occurrence of the components in each latent class shows the importance of these clusters. These findings can help the healthcare professionals in developing prevention programs.**

**Subgrouping of the participants into separated classes might show some relationship between indicator variables that have been unobservable in the variable-centered analysis. We detected that among the students of the fourth latent class, some of cardiometabolic syndrome did not have an important role in clustering them. In other words, these variables (i. e. abdominal obesity, low HDL, high BP, high FBS and obesity) had low probability in the latent class 4.** Boyko et al. studied metabolic syndrome in a latent class analysis designed study (9). Their findings suggest that on the basis of a specific mechanisms, some of the components of metabolic syndrome may correlate with each other independently of other components, and intra-abdominal fat was the most specific component that its presence would increase the chance of metabolic syndrome. This difference could be related to the study place, the age of participants, definition of the Mets and other unknown factors. Anyway there is a need for more studies about the causality of cardiometabolic syndrome components and the cohort design for assessing the latent class analysis by considering time related variables and the related factors and the possible changes in classifying the children. 

The nominal regression analysis showed that after adjusting the effect of other variables, being female decreases the odds of membership in latent class 2 (OR=0.68) and 3(OR=0.52) compared to latent class 1. It has been suggested that males are likely to have higher triglycerides and cholesterol levels and lower HDL levels compared to female gender, specially premenopausal state would result in less lipid profile of plasma (20, 21). Sexual hormone secretion and metabolism are discussed as the possible causes of different lipid metabolism pathways, and the role of insulin in regulating lipid and glucose metabolism is important with regard to genders, since decreased insulin sensitivity after menopause could have a role in elevated concentration of lipid profile of the serum(22-24); however, our information regarding gender differences and its effect of lipid metabolism is limited; since underlying pathways are complex. 

In conclusion** t**he present study shows the co-occurrence of the cardiometabolic syndrome components among Iranian children and adolecsenst by sub grouping of them into four classes. Results indicated that about 20% of the students are in the moderate risk and high risk classes. It seems necessary to design and implement some scientific interventions among this stratum of the population. With regard to our results, it seems necessary to design cohort and longitudinal studies to determine and monitor the incidence rate of cardiometabolic syndrome components and to assess the changes in latent classes of these syndrome components by considering time varying effects and related factors. The findings of this study can be used in focusing the prevention programs on the most important components in reducing the prevalence in this stratum. 

## References

[1] Srivastava AK (2012). Challenges in the treatment of cardiometabolic syndrome. Indian J Pharmacol..

[2] Castro JP, El-Atat FA, McFarlane SI, Aneja A, Sowers JR (2003). Cardiometabolic syndrome: pathophysiology and treatment. Curr Hypertens Rep.

[3] Ogden CL, Carroll MD, Curtin LR, Lamb MM, Flegal KM (2010). Prevalence of high body mass index in US children and adolescents, 2007-2008. JAMA.

[4] Juonala M, Magnussen CG, Berenson GS (2011). Childhood adiposity, adult adiposity, and cardiovascular risk factors. N Engl J Med.

[5] Orio F, Palomba S, Cascella T (2007). Cardiovascular complications of obesity in adolescents. J Endocrinol Invest.

[6] Fitzpatrick SL, Lai BS, Brancati FL, Golden SH, Hill-Briggs F Metabolic syndrome risk profiles among African American adolescents: national health and nutrition examination survey, 2003-2010.

[7] Kelishadi R, Hovsepian S, Djalalinia S, Jamshidi F, Qorbani M (2016). A systematic review on the prevalence of metabolic syndrome in Iranian children and adolescents. J Res Med Sci.

[8] Kahn R, Buse J, Ferrannini E, Stern M (2005). The metabolic syndrome: time for a critical appraisal Joint statement from the american diabetes association and the european association for the study of diabetes. Diabetologia.

[9] Boyko EJ, Doheny RA, McNeely MJ (2010). Latent class analysis of the metabolic syndrome. Diabetes Res Clin Pract.

[10] Chan JC, Cheung JC, Stehouwer CD (2002). The central roles of obesity-associated dyslipidaemia, endothelial activation and cytokines in the metabolic syndrome—an analysis by structural equation modelling. Int J Obes Relat Metab Disord.

[11] Meigs JB (2000). Invited commentary: insulin resistance syndrome? Syndrome X? Multiple metabolic syndrome? A syndrome at all? Factor analysis reveals patterns in the fabric of correlated metabolic risk factors. Am J Epidemiol.

[12] Shen B-J, Todaro JF, Niaura R, McCaffery JM, Zhang J, Spiro III A (2003). Are metabolic risk factors one unified syndrome? Modeling the structure of the metabolic syndrome X. Am J Epidemiol.

[13] Grundy SM, Cleeman JI, Daniels SR (2005). Diagnosis and management of the metabolic syndrome: an American Heart Association/National Heart, Lung, and Blood Institute scientific statement. Circulation.

[14] Lanza ST, Collins LM, Lemmon DR, Schafer JL (2007). PROC LCA: A SAS procedure for latent class analysis. Struct Equ Modeling.

[15] Abbasi-Ghahramanloo A, Soltani S, Gholami A, Erfani M, Yosaee S (2016). Clustering and combining pattern of metabolic syndrome components among Iranian population with latent class analysis. Med J Islam Repub Iran.

[16] Motlagh ME, Ziaodini H, Qorbani M (2017). Methodology and early findings of the fifth survey of childhood and adolescence surveillance and prevention of adult noncommunicable disease: The CASPIAN-V study. Int J Prev Med.

[17] WHO Multicentre Growth Reference Study Group (2006). WHO Child Growth Standards based on length/height, weight and age. Acta Paediatr Suppl.

[18] Arguelles W, Llabre MM, Sacco RL (2015). Characterization of metabolic syndrome among diverse Hispanics/Latinos living in the United States: latent class analysis from the Hispanic Community Health Study/Study of Latinos (HCHS/SOL). Int J Cardiol.

[19] Katakami N, Kaneto H, Matsuhisa M (2008). Clustering of several cardiovascular risk factors affects tissue characteristics of the carotid artery. Atherosclerosis.

[20] Seidell JC, Cigolini M, Charzewska J (1991). Fat distribution and gender differences in serum lipids in men and women from four European communities. Atherosclerosis.

[21] Wang X, Magkos F, Mittendorfer B (2011). Sex differences in lipid and lipoprotein metabolism: it's not just about sex hormones. J Clin Endocrinol Metab.

[22] Milewicz A, Tworowska U, Demissie M (2001). Menopausal obesity–myth or fact?. Climacteric.

[23] Ferrannini E, Balkau B, Coppack SW (2007). Insulin resistance, insulin response, and obesity as indicators of metabolic risk. J Clin Endocrinol Metab.

[24] Magkos F, Wang X, Mittendorfer B (2010). Metabolic actions of insulin in men and women. Nutrition.

